# 1,5-Anhydroglucitol promotes pre-B acute lymphocytic leukemia progression by driving glycolysis and reactive oxygen species formation

**DOI:** 10.1186/s12885-023-10589-9

**Published:** 2023-02-06

**Authors:** Huasu Zhu, Huixian Ma, Na Dong, Min Wu, Dong Li, Linghong Liu, Qing Shi, Xiuli Ju

**Affiliations:** 1 Department of Pediatrics, Qilu Hospital of Shandong University, Jinan, 250012 Shandong Province China; 2 Laboratory of Cryomedicine, Qilu Hospital of Shandong University, Jinan, 250012 Shandong Province China

**Keywords:** Redox homeostasis, Glucose metabolism, Pre-B acute lymphoblastic leukemia, 1,5-anhydroglucitol, Reactive oxygen species

## Abstract

**Background:**

Precursor B-cell acute lymphoblastic leukemia (pre-B ALL) is the most common hematological malignancy in children. Cellular metabolic reorganization is closely related to the progression and treatment of leukemia. We found that the level of 1,5-anhydroglucitol (1,5-AG), which is structurally similar to glucose, was elevated in children with pre-B ALL. However, the effect of 1,5-AG on pre-B ALL was unclear. Here, we aimed to reveal the roles and mechanisms of 1,5-AG in pre-B ALL progression.

**Methods:**

The peripheral blood plasma level of children with initial diagnosis of pre-B ALL and that of healthy children was measured using untargeted metabolomic analysis. Cell Counting Kit-8 assay, RNA sequencing, siRNA transfection, real-time quantitative PCR, and western blot were performed using pre-B ALL cell lines Reh and HAL-01. Cell cycle, cell apoptosis, ROS levels, and the positivity rate of CD19 were assessed using flow cytometry. Oxygen consumption rates and extracellular acidification rate were measured using XFe24 Extracellular Flux Analyzer. The lactate and nicotinamide adenine dinucleotide phosphate levels were measured using kits. The effect of 1,5-AG on pre-B ALL progression was verified using the In Vivo Imaging System in a xenotransplantation leukemia model.

**Results:**

We confirmed that 1,5-AG promoted the proliferation, viability, and intracellular glycolysis of pre-B ALL cells. Mechanistically, 1,5-AG promotes glycolysis while inhibiting mitochondrial respiration by upregulating pyruvate dehydrogenase kinase 4 (PDK4). Furthermore, high levels of intracellular glycolysis promote pre-B ALL progression by activating the reactive oxygen species (ROS)-dependent mitogen-activated protein kinase/extracellular signal-regulated kinase (MAPK/ERK) pathway. Conversely, N-acetylcysteine or vitamin C, an antioxidant, effectively inhibited 1,5-AG-mediated progression of leukemia cells.

**Conclusions:**

Our study reveals a previously undiscovered role of 1,5-AG in pre-B ALL, which contributes to an in-depth understanding of anaerobic glycolysis in the progression of pre-B ALL and provides new targets for the clinical treatment of pre-B ALL.

**Supplementary Information:**

The online version contains supplementary material available at 10.1186/s12885-023-10589-9.

## Background

Acute leukemia is the most common malignant disease in children. Approximately 80% of cases of this leukemia type are lymphocytic and pre-B cell ALL is the predominant type [1]. Attributable to risk-directed treatment, the 5-year event-free survival rate for childhood ALL is over 80% [2]. However, because of metabolic heterogeneity among tumors, the treatment outcomes of children with ALL vary widely. Metabolic reprogramming, including the upregulation of glycolysis and activation of electron transport chains, is widely used in the diagnosis and treatment of ALL. This process is also involved in ALL relapses [3]. In Philadelphia chromosome-positive ALL, the administration of imatinib or related tyrosine kinase inhibitor significantly improved the patient survival rate [4]. Thus, targeted therapy for ALL is an attractive and promising clinical treatment approach.

Tumor cells have the characteristic of reprogramming metabolism to support the rapid proliferation, continuous growth, invasion, and metastasis of tumor cells under harsh environments, such as hypoxia [5]. Metabolic reprogramming includes three categories: glucose metabolism, amino acid metabolism and fatty acid metabolism. Among them, glucose metabolism provides energy for tumor cells and generates other precursors necessary for biosynthesis or special tumor metabolites to activate a series of signaling pathways [6]. The Warburg effect states that tumor cells can rely on relatively inefficient glycolysis to meet their energy needs even under hypoxic conditions [7]. In addition, in recent years, the study of glucose metabolites in the field of leukemia has deepened [8]. In B cell malignancies, serine/threonine-protein phosphatase 2A directs glucose carbon utilization from glycolysis to the pentose phosphate pathway to rescue oxidative stress and maintain the proliferation requirements of B cell malignancies [9]. Additionally, R-2-hydroxyglutarate, a metabolite of mutant isocitrate dehydrogenases, inhibits tumor cell glycolysis and blocks the progression of acute myeloid leukemia (AML) [10]. Therefore, metabolic therapy has become a promising approach for clinical ALL therapy.

Glucose metabolism is closely associated with redox homeostasis, which is an important component of physiological cellular homeostasis [11, 12]. Tumor cells in a highly proliferative state have an active material metabolism, which inevitably leads to an imbalance between redox homeostasis and oxidative stress [13]. Oxidative stress leads to increased levels of reactive oxygen species (ROS), and the effect of ROS on cancer progression is paradoxical. An increase in ROS levels at a specific threshold can promote the growth, metastasis, and angiogenesis of tumor cells. However, ROS production has antitumor effects when it reaches the toxic levels, leading to increased oxidative stress and induction of tumor cell death [14, 15]. This phenomenon shows the complexity of antioxidants in the treatment of cancer. In hematological diseases, ROS production promotes the proliferation of AML by upregulating the expression of a key regulatory glycolytic enzyme, 6-phosphofructo-2-kinase/fructose-2,6-bisphosphatase [16]. Inhibition of ROS limits the malignant expansion of chronic lymphocytic leukemia cells in vivo [17]. Therefore, targeting metabolic and oxidative stress has become a new direction in the treatment of leukemia.

In this study, we aimed to determine the role of 1,5-anhydroglucitol (1,5-AG) in the progression of pre-B ALL. Using untargeted metabolomics, we identified high levels of 1,5-AG metabolites in children with pre-B ALL and confirmed that 1,5-AG promotes the malignant progression of pre-B ALL by regulating glucose metabolism. Mechanistic studies have shown that the pro-tumor activity of 1,5-AG is regulated by the ROS-dependent mitogen-activated protein kinase/extracellular signal-regulated kinase (MAPK/ERK) signaling pathway. Our results revealed a novel metabolite that promoted pre-B ALL progression by driving glycolysis and ROS formation.

## Methods

### Patients and extraction of peripheral blood plasma

Peripheral blood (PB) samples were obtained from 27 children with pre-B ALL at the time of initial diagnosis prior to chemotherapy and 27 healthy donors at the Qilu Hospital of Shandong University from December 2019 to April 2020, with informed consent from the children’s legal guardians. The clinical characteristics of children are presented in Table S1. This study was approved by the Ethics Committee of the Qilu Hospital of Shandong University, Shandong Province (KYLL-202008-176). PB samples were collected before chemotherapy. The samples were immediately stored on ice and transported to the laboratory within 1 h. After centrifugation for 15 min at 1800×g and 4 °C, 500 μL of the plasma was placed in a new asepsis tube, frozen in liquid nitrogen, and stored at − 80 °C until analysis.

### Metabolite extraction and liquid chromatography-mass spectrometry-based metabolomic analysis

For the metabolite extraction, 200 μL of blood plasma was treated with 500 μL ice-cold 80% methanol (precooling at − 80 °C). Then, samples were vortexed 30 s for at least 15 min on ice and centrifuged for 15 min at 16,000×g at 4 °C. The supernatant was transferred to a new asepsis tube, and the samples were dried down on the Integrated SpeedVac System (ISS110-230, Thermo Fisher Scientific, Waltham, MA, USA) at 30 °C. The samples were sealed and stored at − 80 °C. For the quantitative detection of 1,5-AG in mouse plasma, we created an internal standard as follows: Practically, we added 100 μL of plasma to 195 μL of (20% H_2_O/80% methanol) and spiked it with 5 μL of 450 μM 1,5-AG (7.5 μM final concentration) (309,742, Jkchemical, Beijing, China). Metabolite extraction was performed, as previously described.

For mass spectrometry-based analysis of extracted metabolites, patient samples were commissioned by Aksmics Shanghai (Shanghai, China). PB plasma samples were collected from the mice at the Advanced Medical Research Institute, Shandong University (Shandong, China). Liquid chromatography-mass spectrometry (LC-MS) analysis was performed on a C_18_ column (059149, 2.1 mm × 100 mm, 2.2 μm, Thermo Fisher Scientific) using mobile phase A consisting of water mixed with 25 mM ammonium acetate and mobile phase B comprising acetonitrile. The mobile phase profile consisted of the following steps and linear gradients: 0–1 min at 1% B, 1–6 min from 1 to 50%, 6–8 min from 50 to 80%, 8–9 min at 80% B, 9–10 min from 80 to 5%, and 10–12 min at 5% B. The injection volume was 15 μL, and the flow rate was 0.4 mL/min. MS analysis was performed using Q-Exactive MS/MS in the negative ion mode. The absolute concentrations of 1,5-AG were determined using integrated extracted ion chromatograms corresponding to 1,5-AG (m/z = 163.0612). The peak heights were used for data reporting, and the data were normalized using internal standards.

### Cell culture

The human pre-B ALL cell lines Reh and HAL-01 were obtained from Procell Life Science & Technology Co., Ltd. (Wuhan, China) and authenticated using short tandem repeat profiling. Two cell lines were cultured in Roswell Park Memorial Institute (RPMI)-1640 (Gibco, Waltham, MA, USA) containing 10% fetal bovine serum (Gibco) and 1% penicillin-streptomycin (Gibco) at an incubator with a temperature of 37 °C and 5% CO_2_. All cell lines were tested for mycoplasma contamination using the GSDetectTM Taqman Mycoplasma Detection Kit (D0102, Guangzhou Geneseed Biotech Co., Guangzhou, China). In some experiments, the medium was prepared by adding different doses of 1,5-AG, vitamin C (A92902, Sigma Sigma Aldrich, St Louis, MO, USA), and 2 mM N-acetylcysteine (NAC) (A7250, Sigma Aldrich). To investigate the distribution of Reh in vivo, Reh was labeled with mCherry by lentivirus transfection (Shanghai Genechem Co., Ltd., Shanghai China). Subsequently, 1 μg/mL Purinomycin (ST551, Beyotime, Shanghai, China) was added to infected cells to screen for mCherry expression. PB-mononuclear cells (PB-MNCs) were isolated from the whole blood via density gradient centrifugation using Ficoll. CD19-positive cells in PB-MNCs close to or greater than 90% were selected. The PB-MNCs were cultured in RPMI 1640 complete medium for 24 h and, then, treated with different doses of 1,5-AG. Follow-up experiments were performed at 24 h after the intervention.

### Animal procedures

For the “human-in-mouse” xenotransplantation pre-B ALL mice model, mCherry-Reh cells (4 × 10^6^) were transplanted into 5- to 6-week-old male NPI (Beijing IDMO Co., Ltd., Beijing, China) recipient mice intravenously. The mice were then randomly divided into five groups (five mice/group): blank, model, NAC, 1,5-AG (AG for figure), and 1,5-AG + NAC. In brief, 1,5-AG was administered daily by gavage (100 μL of 100 mM solution). To determine the effect of 1,5-AG-induced ROS on leukemic cell growth, we administered an intraperitoneal injection of 75 mg/kg/day NAC or phosphate-buffered saline (PBS) as control daily until the mice were sacrificed on day 4 or 7. Leukemia development and progression were measured using the In Vivo Imaging System (IVIS) Spectrum instrument (PerkinElmer Inc., Waltham, MA, USA). The mice were euthanized by CO_2_ inhalation. The PB, bone marrow (BM), and spleen samples were collected on ice for further analysis. PB plasma was collected for the targeted detection of 1,5-AG metabolites. The animal experiments were approved by the Ethics Committee on Animal Experiment of Shandong University Qilu Hospital (DWLL-2021-035, Shandong, China).

### Flow cytometry

The percentage of CD19^+^ PB-MNCs from PB samples and “human-in-mouse” pre-B ALL model were detected by flow cytometry with APC- or PE-conjugated mouse anti-human CD19 antibody (302,212 and 982,402, Biolegend, San Diego, CA, USA). Flow cytometry was performed using the Guava easyCyte 6HT system (EMD Millipore, Billerica, MA, USA), and the data were analyzed using the Guava Incyte software (3.1 version, EMD Millipore).

### Cell viability assay

Cells (1 × 10^5^/mL) were treated with different doses of 1,5-AG or 2 mM NAC and seeded in 12-well plates. After 12 or 24 h of culture, 100 μL of the cell suspension was sucked out in 96-well plates in triplicate. Cell viability was determined using the Cell Counting Kit-8 (CCK-8) assay (CK04-500 T, Dojindo, Shanghai, China). Subsequently, 10 μL of the CCK-8 reagent was added to each well. After 3 h of continuous culture in an incubator, the absorbance values of the plates were measured at 450 nm using a spectrophotometer (Bio-Rad Laboratories, Hercules, CA, USA) and recorded for analysis.

### Cell cycle and cell apoptosis analyses by flow cytometry

The percentage of cells located at the G0/G1, S, and G2/M phases was assessed using propidium iodide (PI) (550,825, BD Biosciences, Franklin Lakes, NJ, USA). Subsequently, 1 × 10^6^ cells were collected and fixed at 4 °C, washed with PBS twice after 12 h, resuspended in 500 μL PI containing 50 μL RNase A (10 mg/mL) (R1030, Solarbio, Beijing, China), and incubated at 37 °C for 20 min. Then, the cells were examined by flow cytometry. Cell apoptosis was evaluated using the FITC Annexin V Apoptosis Detection Kit (556,547, BD Biosciences) following the manufacturer’s instructions.

### XFe24 Extracellular Flux Analyzer

Oxygen consumption rates (OCR) and extracellular acidification rate (ECAR) were measured using Seahorse XF Cell Mito Stress Test Kit (103015-100, Agilent Technologies, Santa Clara, CA, USA) and Seahorse XF Glycolytic Rate Assay Kit (103344-100, Agilent Technologies), respectively. Seahorse XFe24 Extracellular Flux Analyzer (Agilent) was used for analysis according to the manufacturer’s protocol. Cells were seeded at a density of 6 × 10^5^ cells per well for Reh and 8 × 10^5^ per well for HAL-01. Plates were pre-coated with Cell-Tak™ (354,240, BD Biosciences). Each sample was assayed based on a minimum of three replicates, and the data were normalized to the protein content in each well.

### Measurement of lactate levels

The lactate levels were detected by Lactate Colorimetric Assay Kit (BC2230, Solarbio). Cells (1 × 10^6^) were washed with PBS and lysed with 1 mL lactate extract buffer. Then, 0.8 mL of the supernatant was removed by centrifugation and transferred to a new asepsis tube. Making standard curves according to manufacturer’s recommendations. After incubation with the reaction mixture for 30 min, the insoluble materials were centrifuged and dissolved in ethanol. The samples were measured at 570 nm, and the lactate levels were calculated using a standard curve.

### Measurement of nicotinamide adenine dinucleotide phosphate levels

Cells (1 × 10^6^) were lysed with 200 μL of extract buffer. The nicotinamide adenine dinucleotide phosphate (NADPH) levels in cell lysates were measured using the NADP+/NADPH Assay Kit (S0179, Beyotime) according to the manufacturer’s instructions. After incubation for 30 min, the samples were measured at 450 nm, and the NADPH levels were calculated using the prepared standard curve.

### Detection of reactive oxygen species (ROS)

Cells (1 × 10^5^/mL) were seeded in six-well plates with different doses of 1,5-AG. After culturing for 24 h, cells were washed twice and resuspended in serum-free medium containing 10 μM of fluorescent probe dichloro-dihydro-fluorescein diacetate (DCFH-DA) (S0033S, Beyotime) and stored at 37 °C for 20 min in the dark. Then,the cells were washed with PBS and analyzed by flow cytometry.

### RNA-seq experiments

Cells were immediately collected for RNA extraction using TRIzol (T9424-100 mL; Sigma Aldrich). All RNA sequencing samples were commissioned and analyzed by Xiuyue Biol (Jinan, China). The original data are available in the [GEO] repository.

### siRNA transfection

Cells were transfected with siRNA targeting pyruvate dehydrogenase kinase 4 (PDK4) (siPDK4, GenePharma, Shanghai, China) or negative control (siNC, GenePharma) using Lipofectamine® 2000 (11,668,500, Invitrogen, Waltham, MA, USA). The sequences of the siRNAs were as follows: siPDK4-1 (CAACGCCUGUGAUGGAUAA), siPDK4-2 (GACCGCCUCUUUAGUUAUA), siNC (UUCUCCGAACGUGUCACGU).

### RNA extraction and real-time quantitative PCR (RT-qPCR) analysis

Total RNAs were extracted with RNAfast2000 assay Kit (220,011, Fastagen, Shanghai, China). For cDNA synthesis, 1 μg of total RNA was used in 10 μL of reverse transcriptase reaction mixture using the ReverTra Ace qPCR RT Master Mix kit (FSQ-201, TOYOBO, Osaka, Japan) with specific primers. RT-qPCR was performed using a Real Time Thermo cycler (Analytik Jena AG, qTOWER3G, Germany), and detection was performed with SYBR Green Realtime PCR Master Mix (QPK-201, TOYOBO, Osaka, Japan) in a 20 μL reaction mixture to detect the mRNA levels of cytokines. The primer sequences used in the RT-qPCR analysis were as follows: PDK4 forward: 5′-GGTGGTGTTCCCCTGAGAAT-3′; PDK4 reverse: 5′-GCAAGCCGTAACCAAAACCA − 3′. The data were normalized to the amount of the glyceraldehyde 3-phosphate dehydrogenase (GAPDH) transcript. Each reaction was run in triplicates, and the mRNA levels were normalized to the level of the Control group as reference, which was set to 1.

### Western blotting analysis

For total protein extraction, the cells were washed with PBS, lysed in Radio-Immunoprecipitation Assay buffer (R0020, Solarbio) with phosphatase/protease inhibitor (P1005, Beyotime), and measured by ultraviolet spectrophotometry. The lysates were separated by 10% sodium dodecyl sulfate-polyacrylamide gel electrophoresis and then transferred onto a polyvinylidene fluoride membrane. The membranes were blocked with 5% bovine serum albumin and incubated with primary antibodies raised against GAPDH (1:1000; 5174S, Cell Signaling Technology, Danvers, MA, USA), PDK4 (1:500, 12,949-1-AP, Proteintech, Rosemont, IL, USA), p44/42 MAPK (ERK1/2) (1:2000, 4695S, Cell Signaling Technology), and phospho-p44/42 MAPK (ERK1/2) (Thr202/Tyr204) (1:2000, 4370S, Cell Signaling Technology). Goat anti-rabbit antibodies conjugated to horseradish peroxidase were used as secondary antibodies (1:5000; ZB-2301, ZSGB-BIO, Beijing, China), and the proteins were visualized using enhanced chemiluminescence (JIAPENG, JP-K600plus, Shanghai, China). The band intensity was quantified using the ImageJ software (US National Institute of Health, Bethesda, NC, USA). The experiments were performed in triplicate.

### Immunohistochemical (IHC) staining

The IHC staining was carried out as described previously [18]. The antibodies used in this study were phospho-p44/42 MAPK (ERK1/2) (Thr202/Tyr204) (1:500, 4370S, Cell Signaling Technology). The slides were observed under a microscope (ECHO, RVL2-K) and photographed (ECHO Pro v6.3, Lake Zurich, IL, USA). Protein expression was quantitated by integral optical density (IOD) using ImageJ software.

### Statistical analyses

GraphPad Prism 8.0.1 (GraphPad Software, Inc., La Jolla, CA, USA) was used for statistical analyses. For the analysis of non-targeted metabolomics, orthogonal partial least squares discriminant analysis (OPLS-DA) was subsequently applied. All data were tested for normality using the Kolmogorov–Smirnov test. The Bonferroni test was performed between groups. Two-tailed unpaired Student’s t tests were used for all comparisons between two groups. Two-way ANOVA with Tukey’s test was used for analysis of different interventions in the presence of 1,5-AG. One-way ANOVA with Dunnett’s test was used to compare different 1,5-AG concentrations, and results with *P* values less than 0.05 were considered significant. All data are presented as means ± standard deviations, and each experiment was biological repeated three times.

## Results

### Metabolite profile of the peripheral blood plasma of children with pre-B ALL

**Fig. 1:**
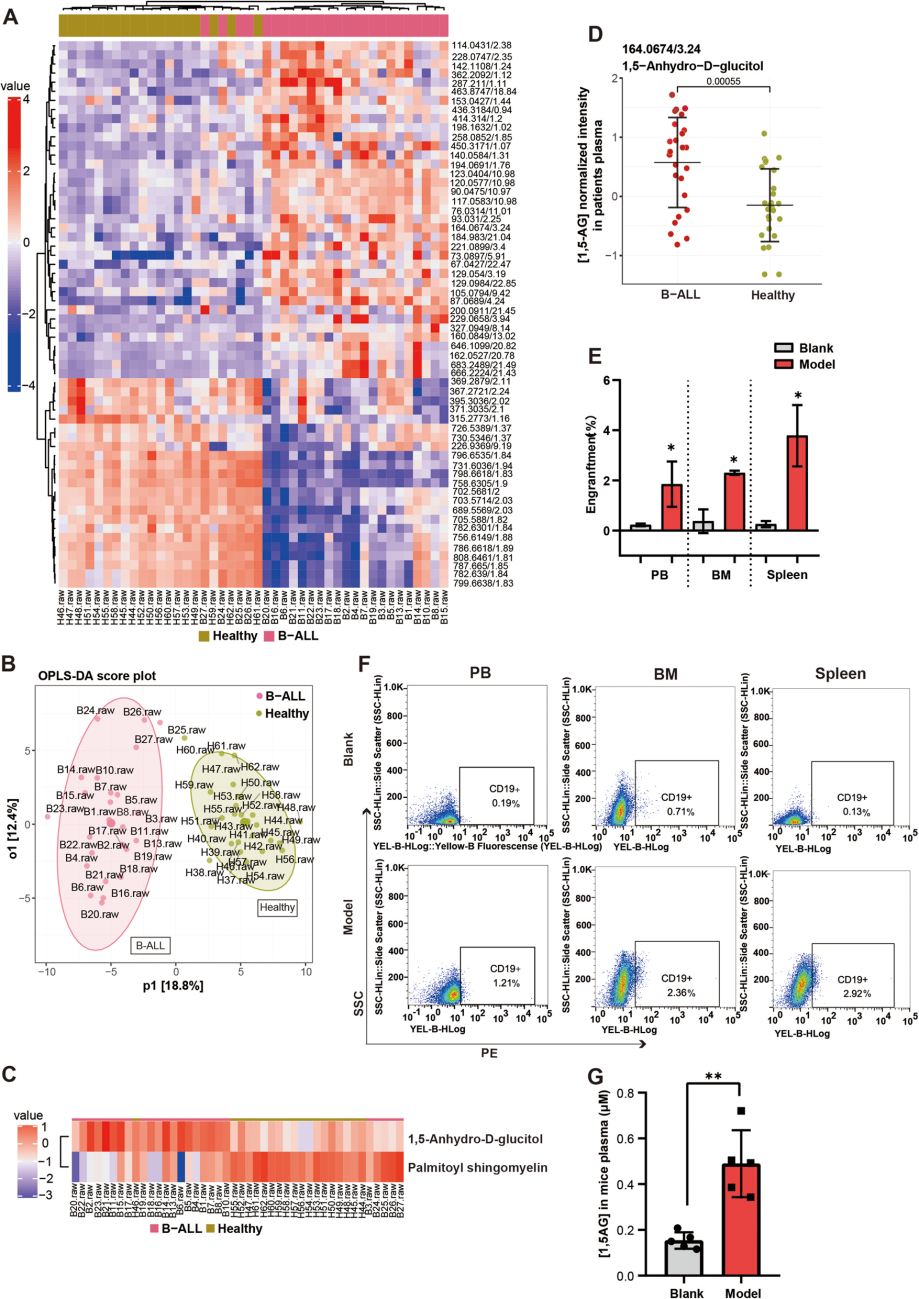
The level of 1,5-anhydroglucitol (1,5-AG) in patients with precursor B-cell acute lymphocytic leukemia (pre-B ALL) and mice model was higher. **A** Cluster heat map shows the significantly elevated and decreased metabolites. **B** Orthogonal partial least squares discriminant analysis for the samples from peripheral blood plasma of patients (*n* = 27) and healthy controls (*n* = 27). **C–D** The exact metabolites of 1,5-AG were significantly increased. **E-F** Engraftment of Reh cells into the peripheral blood (PB), bone marrow (BM), and spleen on pre-B ALL mice model or blank (*n* = 5). **G** 1,5-AG was also elevated in the peripheral blood of the pre-B ALL mice model (*n* = 5). Data are represented as means ± standard deviations. ^*^P < 0.05, ^**^P < 0.01

To seek for efficient metabolite-associated therapeutic targets in pre-B ALL, a non-targeted metabolomic analysis was performed for 37 upregulated and 23 downregulated metabolites (Fig. 1A). The results of OPLS-DA showed that the metabolites in the peripheral blood plasma of patients with pre-B ALL were different from those of healthy children (Fig. 1B). The blood plasma levels of 1,5-AG were approximately one to two times higher in patients with pre-B ALL than in healthy children (Fig. 1C, D). Further, we constructed a “human-in-mouse” pre-B ALL model, and the proportion of anti-human CD19^+^ cells in the PB, BM, and spleen in the model group increased (Fig. 1E, F). Moreover, 1,5-AG was also higher in the model than in the blank group (Fig. 1G, Fig. S1A). These results indicate that 1,5-AG levels are generally elevated in pre-B ALL, suggesting that 1,5-AG may play a role in pre-B ALL.

### 1,5-Anhydroglucitol promotes pre-B ALL cells proliferation in vitro by enhancing glycolysis and oxidative stress

**Fig. 2:**
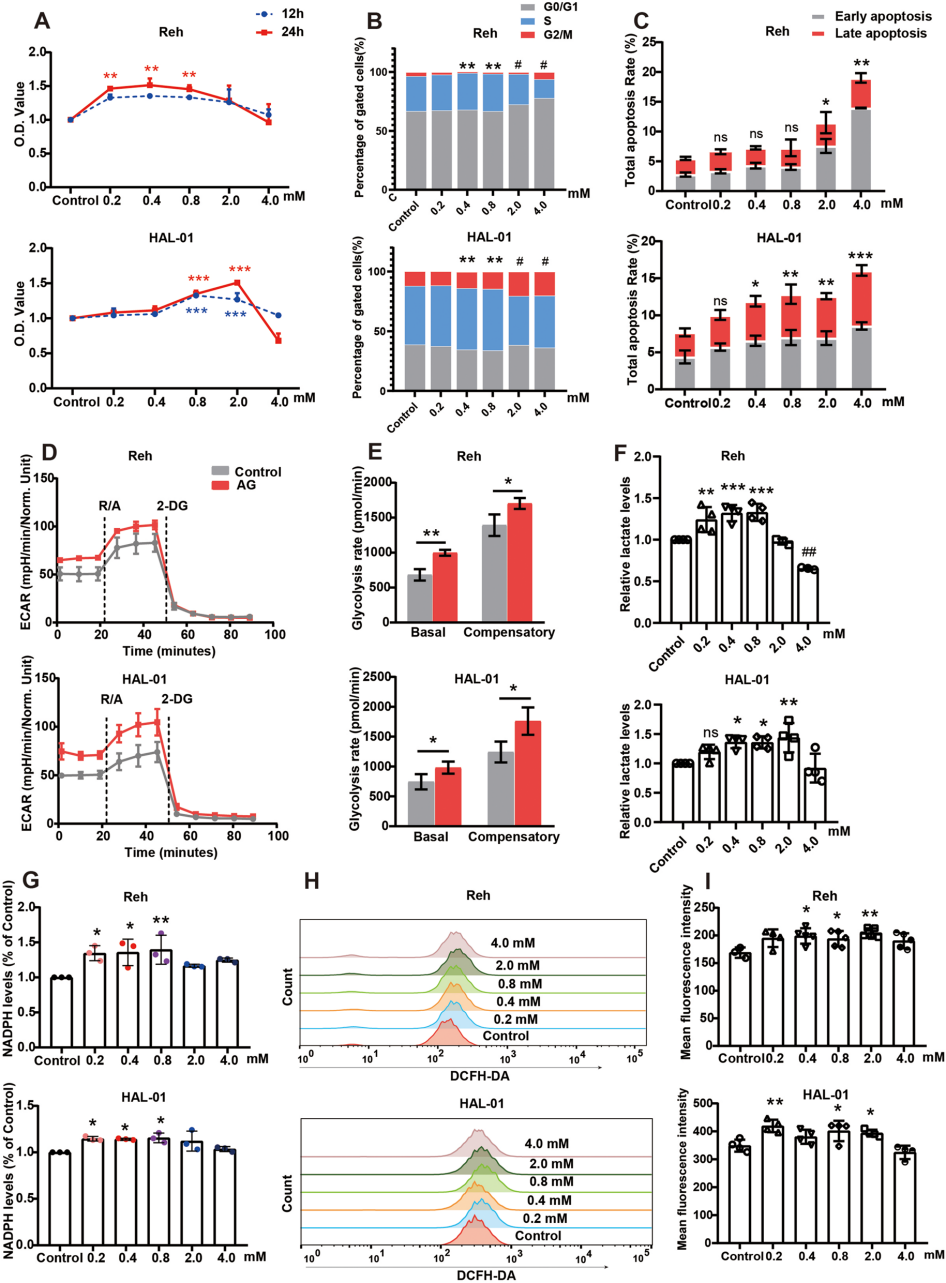
1,5-AG promoted pre-B ALL cell lines proliferation by enhancing glycolysis and oxidative stress. **A** The effects of different pre-B ALL cell lines treated with different doses of 1,5-AG for the indicated times on cell viability. **B** Effects of 1,5-AG on the cell cycle in pre-B ALL cell lines for 24 h. **C** Effects of 1,5-AG on apoptosis in pre-B ALL cell lines for 24 h. **D–E** Effects of 0.8 mM 1,5-AG on glycolytic rates in pre-B ALL cell lines, as determined by the Seahorse Glycolytic Rate Assay. **F** The lactate levels were used to verify the effect of 1,5-AG on cellular glycolysis when the pre-B ALL cell lines were treated with different doses of 1,5-AG for 24 h. **G** Effects of 1,5-AG on nicotinamide adenine dinucleotide phosphate levels in pre-B ALL cell lines for 24 h. **H-I** Reh cells and HAL-01 cells were treated with 1,5-AG, and ROS-positive cells were then measured and analyzed by flow cytometry. Data are presented as means ± standard deviations. ns (*P* ≥ 0.05), ^*^P < 0.05, ^#^*P* < 0.05, ^**^P < 0.01, ^##^*P* < 0.01, ^***^P < 0.001

Referring to the results of metabolite profile, we set the concentration gradient of 1,5-AG in vitro (0.2 mM as physiological normal concentration) (Fig. 2). To assess the effects of 1,5-AG on pre-B ALL cells, cell viability was detected by CCK-8 assay. As shown in Fig. 2A, the addition of 0.2–0.8 mM and 0.8–2.0 mM 1,5-AG at 24 h significantly promoted cell viability in Reh and HAL-01 respectively. However, 1,5-AG did not cause cell proliferation when the concentration reached 2 mM (for Reh) or 4 mM (for HAL-01). The dose-dependent (two to four times the normal concentration) promotion effect of 1,5-AG on pre-B ALL cells was also evident in the cell cycle assay (Fig. 2B). Next, we examined the 1,5-AG-mediated cell cycle progression in pre-B ALL cells. Compared with the control group, the 1,5-AG concentrations of 0.4 mM and 0.8 mM cells were increased at S phase (^**^P < 0.01). Cell cycle arrest was observed at the G2/M phase with 2–4 mM (^#^*P* < 0.05). It is worth mentioning that 1,5-AG had a dose-dependent effect on the apoptosis of HAL-01 (Fig. 2C). Our results suggest that 1,5-AG promotes the viability of pre-B ALL cells by increasing the S phase of cells.

In B-ALL cells, rapid uptake of glucose is accompanied by an increase in glycolysis, which causes oxidative stress in tumor cells [18, 19]. To verify whether 1,5-AG, a glucose analog, affects the rate of glycolysis, we measured the glycolytic rates in pre-B ALL cells. Consistently, the addition of 1,5-AG significantly improved both the basal and compensatory glycolytic rates and increased the lactate levels in both Reh and HAL-01 cells (Fig. 2 D–F). These results indicate that 1, 5-AG is closely related to glycolysis. To further investigate the effect of glycolysis on intracellular oxidative stress, we measured the NADPH levels, and the results indicated that the amount of NADPH produced by 1,5-AG was significantly higher than that produced by the control cells (Fig. 2G). The simultaneous production of NADPH oxidases is a major source of intracellular ROS [20]. Furthermore, we measured the cellular ROS levels in pre-B ALL cells treated with different concentrations of 1,5-AG for 24 h using the fluorescent probe DCFH-DA. Expectedly, 1,5-AG (0.2–0.8 mM) increased the cellular ROS levels in pre-B ALL cell lines (Fig. 2H, I). Taken together, our data indicate that 1,5-AG can promote the proliferation of pre-B ALL cells by enhancing glycolysis, which is essential to induce oxidative stress.

### PDK4 is required for the effects of 1,5-AG on glucose metabolism in pre-B ALL cells

**Fig. 3:**
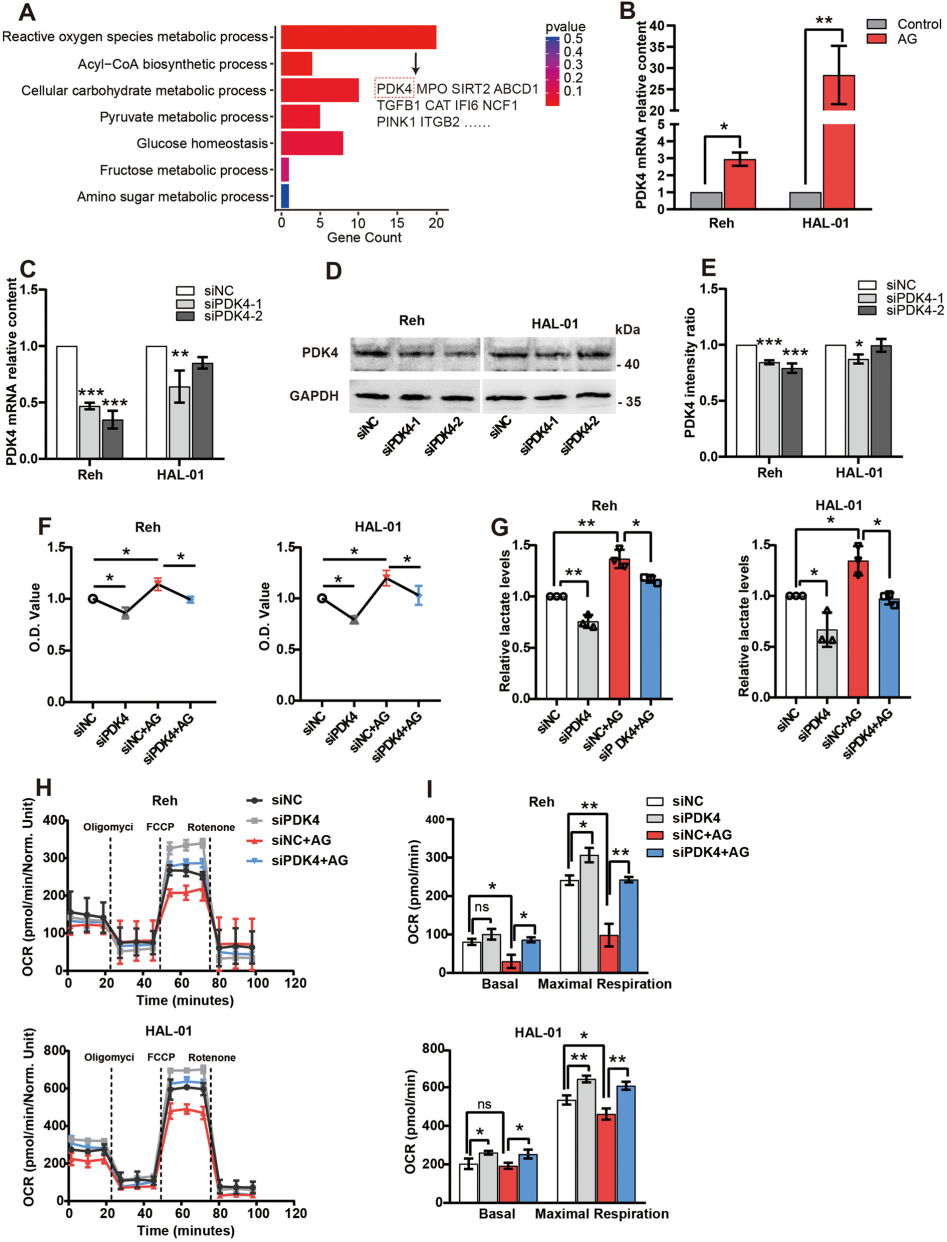
1,5-AG upregulated pyruvate dehydrogenase kinase 4 (PDK4) expression to promote anaerobic glycolysis in pre-B ALL cells. **A** Biological processes enrichment pathways diagram. Pathways are arranged by their *P* value. **B** Effects of 1,5-AG on *PDK4* mRNA expression were determined by real-time polymerase chain reaction (RT-qPCR). **C** RT-qPCR performed to detect PDK4 mRNA in control (siNC) **D-E** Western blot performed to detect protein in control (siNC). Original blots are presented in Supplementary Information 2. **F** Effect of siPDK4 on cell viability (**G**) Effect of siPDK4 on lactate levels. **H-I** The oxygen consumption rate was measured by using a Seahorse XF Analyzer. The final concentration of 1,5-AG was 0.8 mM. Data are presented as means ± standard deviations. ^*^P < 0.05, ^**^P < 0.01, ^***^P < 0.001

To further explore the mechanism by which 1,5-AG enhances pre-B ALL glycolysis, we performed transcriptomic analysis on HAL-01. Gene ontology analysis showed that the differentially expressed genes were significantly enriched in ROS and carbohydrate metabolism processes (Fig. 3A). Among them, *PDK4* was highly expressed and confirmed in pre-B ALL cell lines by RT-qPCR (Fig. 3B). PDK4 regulates glucose metabolism by regulating pyruvate dehydrogenase and reducing the conversion of pyruvate to acetyl-CoA [21]. To determine whether PDK4 mediates the glucose metabolism effect of 1,5-AG, we knocked down PDK4 with siRNAs in pre-B ALL. Two siRNAs targeting PDK4 were used, and the protein levels in pre-B ALL were determined by RT-qPCR and western blot. Selected siPDK4-1 were kept for subsequent experiments (Fig. 3C-E). First, we found that knockdown of PDK4 alone significantly reduced cell viability and lactate levels. Next, knockdown of PDK4 also reduced cell viability and lactate levels of pre-B ALL compared with siNC-transfected cells to which 1,5-AG was added (Fig. 3F, G). Moreover, the addition of 1,5-AG significantly decreased cellular mitochondrial maximum respiration by Seahorse XF Analyzer but did not decrease the glycolysis rate. Furthermore, the knockdown of PDK4 reversed this phenomenon (Fig. 3H, I). These data suggest that 1,5-AG enhances anaerobic glycolysis in pre-B ALL cells by interrupting mitochondrial respiration through the upregulation of PDK4.

### 1,5-AG mediates pre-B ALL cells proliferation by a reactive oxygen species dependent mechanism

**Fig. 4:**
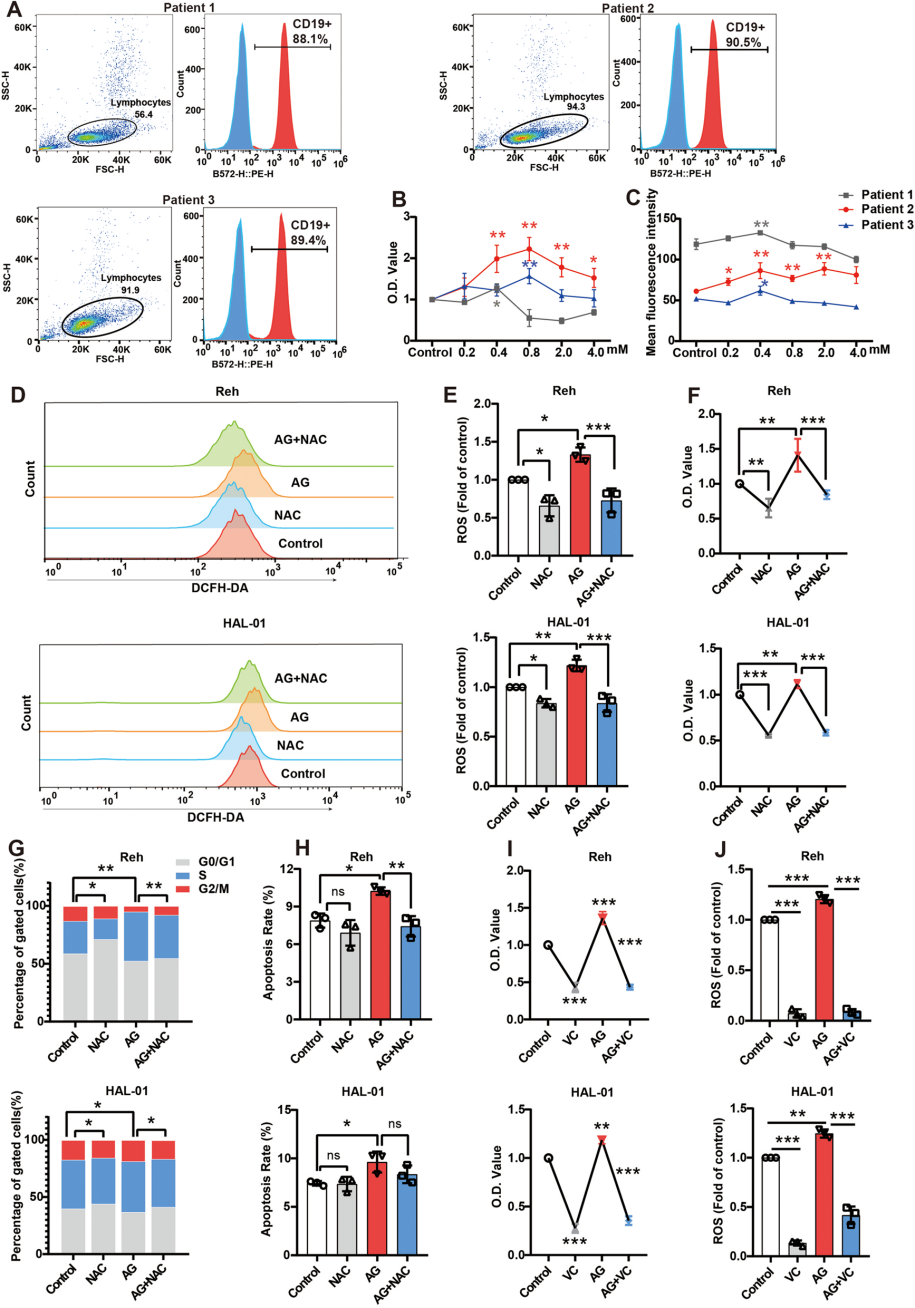
1,5-AG promotes pre-B ALL cells proliferation by inducing cellular reactive oxygen species (ROS) production. **A** The proportion of CD19^+^ cells from peripheral blood-mononuclear cells (PB-MNCs) in three patients with pre-B ALL was detected by flow cytometry. **B** PB-MNCs were treated with different doses of 1,5-AG for 24 h to determine the cell viability. **C** ROS levels detected after 1,5-AG intervention for 24 h. **D–E** Cells were treated with 0.8 mM 1,5-AG with 2 mM NAC, and the ROS-positive cells were then measured and analyzed by flow cytometry. **F–H** Effects of 0.8 mM 1,5-AG with 2 mM NAC on cell viability (**F**), cell cycle (**G**), and apoptosis (**H**) in Reh and HAL-01 cells. **I-J** Effects of 0.8 mM 1,5-AG with 0.2 mM Vitamin C on cell viability (**I**) and ROS levels (**J**). Data are represented as means ± standard deviations. ns (*P* ≥ 0.05), ^*^P < 0.05, ^**^P < 0.01, ^***^P < 0.001

Next, we verified these results using three patient-derived pre-B ALL samples. The positivity rate of CD19 in all three patients’ samples was close to 90% (Fig. 4A). CCK-8 results showed that 1,5-AG increased cell viability in Patient 1 (using concentration of 0.4 mM) and Patient 3 (using concentration of 0.8 mM), and the cell viability of Patient 2 was dose-dependent under 0.4–2.0 mM 1,5-AG (Fig. 4B). The level of ROS increased similarly in the pre-B ALL patient samples (Fig. 4C).

Moreover, the 1,5-AG-induced elevation of ROS levels was effectively reversed by 2 mM NAC (an ROS scavenger) treatment (Fig. 4D, E). Meanwhile, the promoting effect (including an increase in cell viability, cell cycle progression, and induction of cell apoptosis) could be reversed by treatment with NAC in pre-B ALL cells (Fig. 4F–H). Vitamin C, another antioxidant, was also able to reverse the 1,5-AG-induced promoting effect (Fig. 4I, J and Fig. S2). Collectively, our results support the pro-tumor activity of 1,5-AG by ROS-dependent through forward and reverse experiments.

### ROS scavenger can reverse 1,5-AG-induced leukemogenesis in vivo

**Fig. 5:**
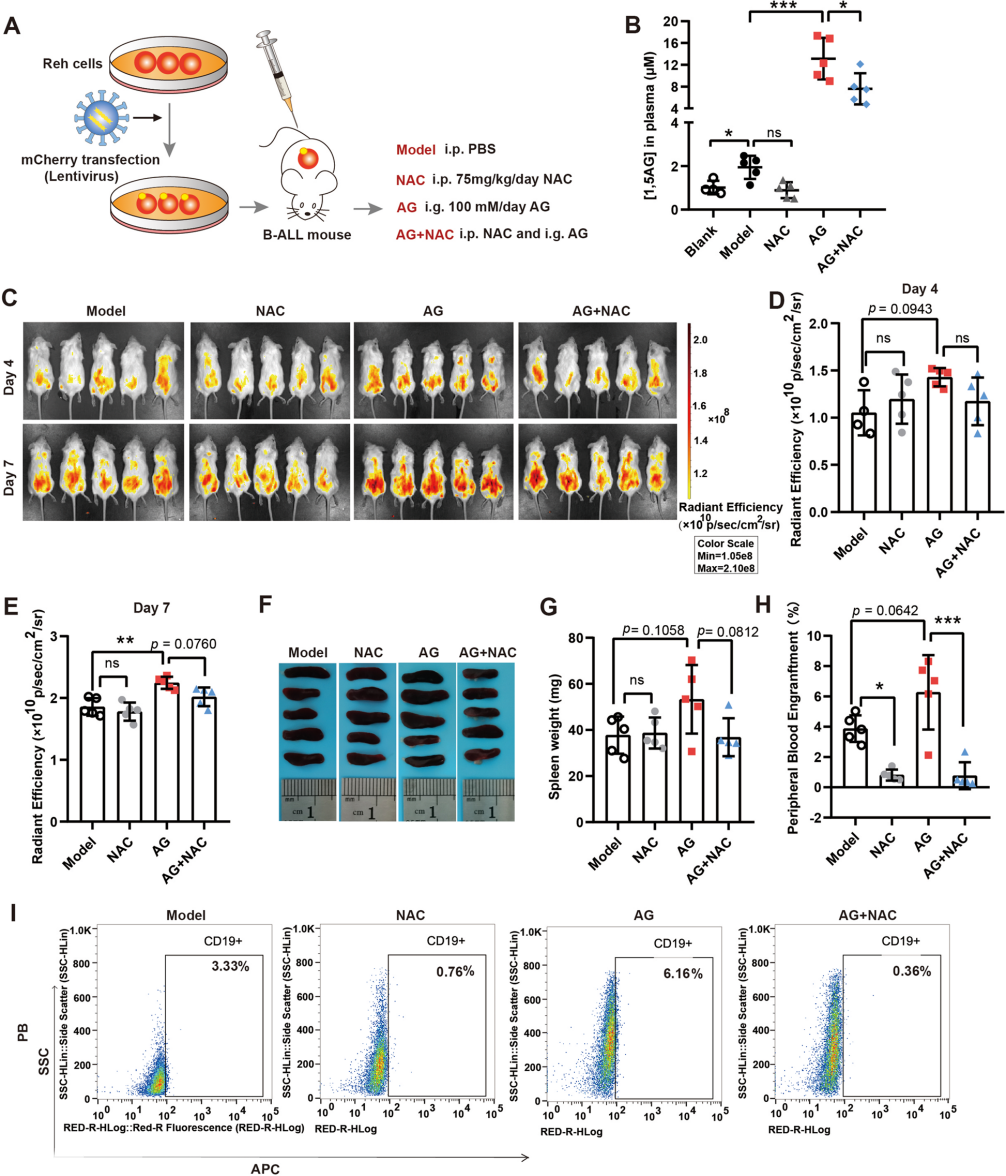
1,5-AG promoted leukemic progression in a xenotransplantation mouse model. **A** Schematic representation of the experimental design process and grouping. **B** Detection of 1,5-AG in the peripheral blood of mice on day 7 in different groups was performed by liquid chromatography-mass spectrometry. **C** In vivo leukemia burden *in* mice from different groups using the In Vivo Imaging System (IVIS). **D–E** Statistical analysis of IVIS in different groups of mice using GraphPad software. **F–G** Pre-B ALL mice were grouped and treated with 1,5-AG or N-acetylcysteine. Photographs of the spleen of each mouse in the different groups on day 7 are shown, and weights were statistically analyzed. **H–I** Infiltration of leukemia cells in the peripheral blood for different groups on day 7 was analyzed by flow cytometry. Data are presented as means ± standard deviations (*n* = 5/group). ns (*P* ≥ 0.05), ^*^P < 0.05, ^**^P < 0.01, ^***^P < 0.001

To confirm the role of 1,5-AG in vivo, we constructed “human-in-mouse” xenotransplantation leukemia model. The experimental grouping and process are illustrated in Fig. 5A. The mice were sacrificed 7 days after the gavage of 1,5-AG. The 1,5-AG levels in the PB of mice in each group were detected by LC-MS. As shown in Fig. 5B, the 1,5-AG levels in the model group mice were higher than those in the blank group, as expected. Moreover, the levels of 1,5-AG were gradually upregulated (three to four times higher) in the AG-treated groups. The effect of the ROS scavenger was also demonstrated at the NAC treatment mouse plasma level. The identification of 1,5-AG by LC-MS is presented in Fig. S1B.

The mouse leukemia burden was tracked using the IVIS Spectrum instrument on days 4 and 7 (Fig. 5C). However, there was no statistical difference between the groups on day 4 (Fig. 5D). Compared with the model, the groups treated with 1,5-AG significantly promoted leukemogenesis on day 7 (**P < 0.01), and NAC to a certain extent abolished this effect (*P* = 0.0760) (Fig. 5E).

In addition, the spleen weight of the AG group was higher than that of the model group (Fig. 5F, G, *P* = 0.1058). Consistently, we used flow cytometry to measure the leukemia burden in mice, and the results showed that 1,5-AG improved the proportion of leukemic cells in the PB (Fig. 5H, I, *P* = 0.0642), and BM (Fig. S3A) and increased leukemia cell infiltration into the spleen (Fig. S3B). These findings also suggest that NAC slowdown 1,5-AG induces leukemic progression. Thus, our current findings reveal that 1,5-AG plays a pro-tumor role in pre-B ALL.

### 1,5-AG activates ROS-dependent-manner MAPK/ERK pathway in pre-B ALL cells

**Fig. 6:**
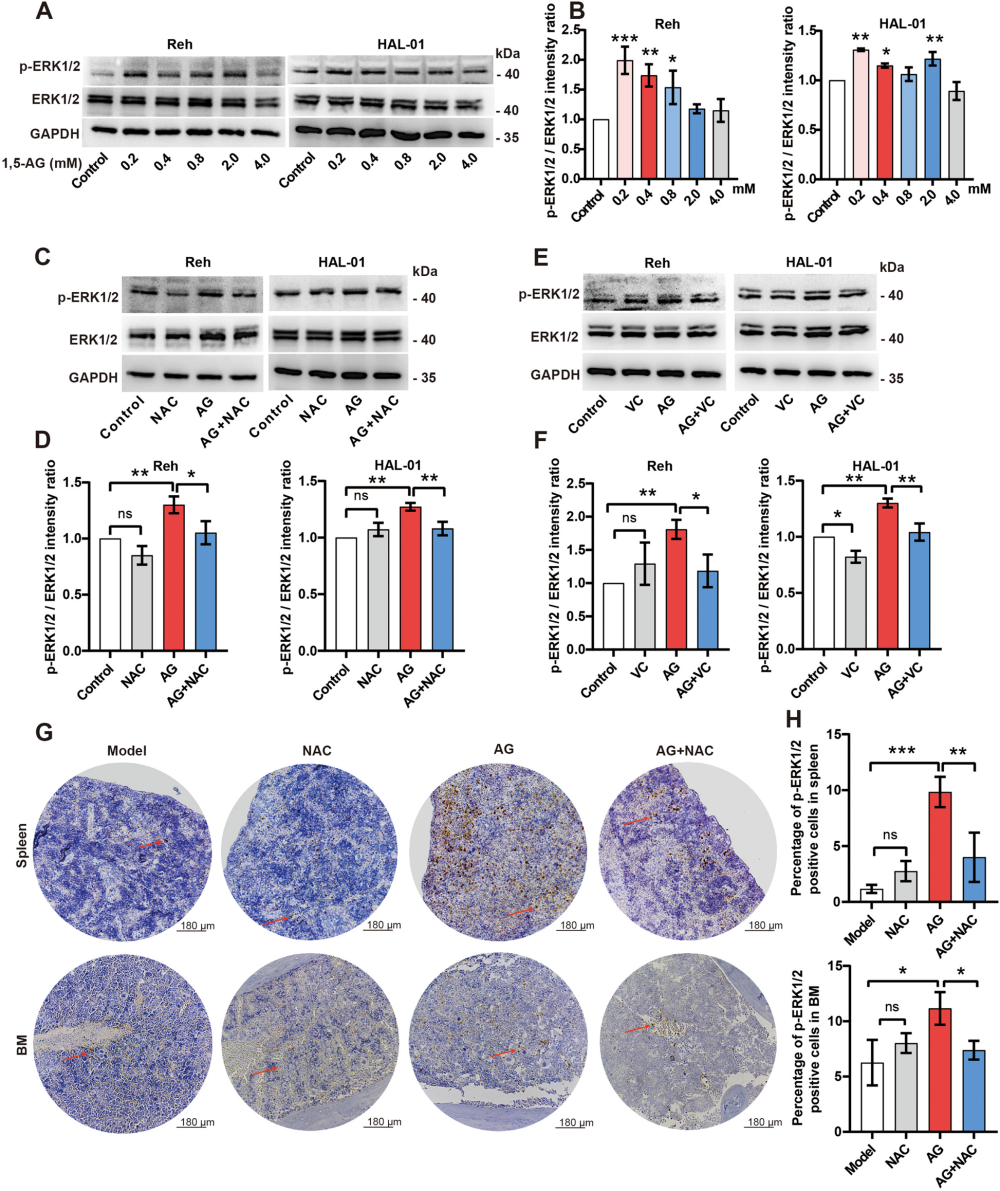
ROS-dependent activation of the MAPK/ERK pathway by 1,5-AG in pre-B ALL. Western blotting (**A**) and statistical analyses (**B**) of p-ERK1/2 and ERK1/2 expressions of pre-B ALL cell lines with different doses of 1,5-AG. **C–F** p-ERK1/2 and ERK1/2 expressions were reversed by NAC and vitamin C treatment in pre-B ALL cells. The final concentration of 1,5-AG was 0.8 mM. Glyceraldehyde 3-phosphate dehydrogenase was used as a loading control. Original blots are presented in Supplementary Information 2. **G–H** The images present p-ERK1/2 staining in the spleen and BM from pre-B ALL mice with the indicated treatments (*n* = 5/group). Scale bars: 100 μm. Data are represented as means ± standard deviations. ns (*P* ≥ 0.05), ^*^P < 0.05, ^**^P < 0.01, ^***^P < 0.001

ROS play an important role in promoting cell proliferation, increasing cell survival and cancer development by regulating the MAPK/ERK pathway [15]. Therefore, we explored the effect of 1,5-AG on the activation of the MAPK/ERK pathway in pre-B ALL cells. As shown in Fig. 6, 1,5-AG (0.2–0.8 mM) markedly increased phosphorylation of ERK1/2 and improved the activity of MAPK/ERK signaling in pre-B ALL cells (Fig. 6A, B). The inhibitory effect of 1,5-AG on the MAPK/ERK pathway was reversed by NAC and Vitamin C treatment in both Reh and HAL-01 cells (Fig. 6C–F). We also assessed the effect of 1,5-AG and NAC in mice by performing p-ERK1/2 staining (Fig. 6G). The results showed that 1,5-AG upregulated p-ERK1/2 positive cells in spleen and BM of mice and this effect was also reversed by NAC (Fig. 6H). These results suggest that 1,5-AG activates the MAPK/ERK pathway in an ROS-dependent manner.

## Discussion

Although the overall survival of pre-B ALL has been greatly improved, the side effects of chemotherapy-based treatment on children are still experienced [22]. Changes in metabolites exist throughout the process of ALL occurrence, development, treatment, and recurrence. Glucocorticoids have been used for more than 50 years as a central component of therapy. Glucocorticoid-mediated reduction of de novo nucleotide synthesis and abnormal lipid metabolism are prevalent in B-ALL chemotherapy [23, 24]. Therefore, it is crucial to find therapeutic targets that act on metabolic processes.

1,5-AG, also known as 1,5-anhydrod-sorbitol, is a six-carbon monosaccharide structurally similar to glucose. In humans, the normal reference values for 1,5-AG are 15.8–52.6 and 14.3–48.0 μg/mL for men and women, respectively [25]. 1,5-AG is widely present in the diet and mainly includes soybean, bread, and rice. After dietary intake, 1,5-AG is rapidly absorbed in the small intestine and excreted quickly into the urine [26]. In addition, the ingestion of less than 20 g of 1,5-AG does not cause side effects in healthy adults [27]. Currently, functional studies on 1,5-AG have mainly focused on the cardiovascular and endocrine systems. Low levels of 1,5-AG correlate with the severity of coronary artery disease [28]. The risk of major adverse cardiac and cerebrovascular events significantly increased when 1,5-AG is < 10.0 μg/mL [29]. Simultaneously, 1,5-AG can reflect the overall glycemic control between 1 day to 1 week, and compensate for hemoglobin A1c deficiency [30]. The cellular metabolism of 1,5-AG has not been fully elucidated. The accumulation of 1,5-AG-6-phosphate, an intracellular metabolic byproduct of 1,5-AG, leads to neutropenia and neutrophil dysfunction, which has been confirmed in clinical trials [31, 32]. However, 1,5-AG in relation to cancer has not been extensively studied [33]. Whether it is involved in regulating the disease development of B-ALL has not been reported. Our results confirmed that 1,5-AG levels were elevated in both patients with pre-B ALL and a mouse model, suggesting that 1,5-AG plays an important role in leukemic progression.

In this study, we found that low concentrations of 1,5-AG are sufficient to effectively regulate glucose metabolism by increasing cellular anaerobic glycolysis. The pyruvate dehydrogenase complex regulates glucose utilization and fatty acid metabolism by controlling the conversion of pyruvate to acetyl-CoA [34]. PDK4, one of the pyruvate dehydrogenase kinases isoenzymes, is present in high-energy-demanding tissues. It works by inactivating the pyruvate dehydrogenase complex [21]. Here, we found that inhibition of anaerobic glycolysis and promotion of mitochondrial respiration by siPDK4 reversed cell viability and reduced the lactate levels. As a glucose-like substance, 1,5-AG may play an oncogenic role in leukemia by regulating glycolysis.

In malignant tumors, glucose metabolism often leads to intracellular oxidative stress and increases ROS levels. Leukemia is no exception and is hypothesized to strongly affect the function of hematopoietic cells. More than 60% of patients with de novo AML have high levels of ROS production, which drives AML cell proliferation through reduced glutathione levels and depletion of antioxidant defense proteins [35]. Moreover, a mice model confirmed that the ROS scavenger NAC restricted the expansion of chronic lymphocytic leukemia cells [17]. In T-cell ALL, interleukin-7 activates the phosphoinositide 3-kinase/Akt/mammalian target of rapamycin pathway and mediates ROS-dependent T cell proliferation and growth [36]. Our current findings strongly suggest that pre-B ALL cell lines undergo ROS-dependent cell proliferation in response to exogenous 1,5-AG. The results were validated in PB-MNC samples from three patients with pre-B ALL. Moreover, the rescue experiments suggest that antioxidant NAC or vitamin C plays an important role in pre-B ALL by 1,5-AG. We demonstrated that the effect of 1,5-AG on pre-B ALL progression is ROS-dependent.

ROS overproduction is a common feature of cancer. In leukemia, although ROS can cause DNA damage, it also promotes the infiltration and proliferation of leukemia cells [37]. In our study, with an increase in 1,5-AG concentration, the level of ROS in the pre-B cell lines increased, causing a certain proportion of apoptosis. We hypothesized that this was DNA damage caused by elevated ROS, and that the cell membrane was slightly outward. Moreover, an increase in the ROS levels can promote the proliferation of tumor cells and is related to the activation of the MAPK/ERK signaling pathway [15, 38]. We investigated the effect of 1,5-AG on this pathway to elucidate the mechanism of promoting pre-B ALL proliferation. The results showed that 1,5-AG significantly upregulated the MAPK/ERK pathway through the ROS-dependent activation of ERK1/2 phosphorylation levels. In addition to directly promoting tumor proliferation, ROS act as signaling molecules to mediate various growth-related responses. Vascular endothelial growth factor (VEGF) stimulates tube formation and proliferation by regulating the angiogenic growth factor VEGF [39]. ROS also serve as secondary messengers to activate the expression of tumor metastasis-related proteases, leading to the aggravation of tumor metastasis [40]. A similar mechanism exists for pre-B ALL. Combined with the pre-B ALL mouse model, our data showed that NAC suppressed 1,5-AG-induced leukemia cell infiltration by tracking leukemia burden with flow cytometry. Overall, 1,5-AG, similar to an oxidant, plays a critical tumor-promoting role in pre-B ALL.

**Fig. 7:**
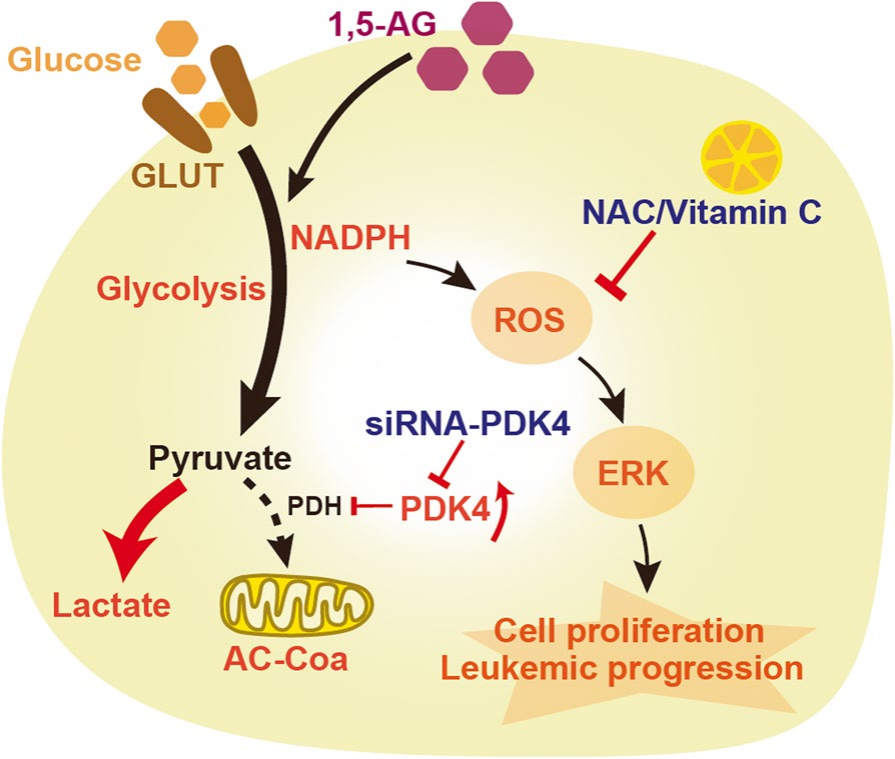
The potential role of 1,5-AG in the progression of pre-B acute lymphocytic leukemia. Enhanced anaerobic glycolysis of pre-B ALL cells was observed with 1,5-AG by promotion of cell viability and cell cycle, and increased lactate accumulation by upregulation of PDK4. Meanwhile, the elevated levels of glycolysis increased the levels of intracellular ROS, which in turn activated the MAPK/ERK pathway, thus, promoting the progression of leukemia. Both siPDK4 and the treatment of antioxidants were effective in the prevention of 1,5-AG-mediated malignant progression of pre-B ALL

Through in vitro and in vivo studies, we concluded that 1,5-AG may accelerate pre-B ALL proliferation by promoting anaerobic glycolysis through upregulation of PDK4. Increased levels of oxidative stress and activation of the ROS-dependent MAPK/ERK pathway also contribute to this acceleration. Antioxidant treatment was effective in blocking the progression of pre-B ALL (Fig. 7). However, there are some limitations in this study, which only detected the 1,5-AG in the PB of children with initial diagnosis pre-B ALL. The dynamic detection of 1,5-AG abundance in the treatment, prognosis, and recurrence of pre-B ALL needs further research and analysis.

## Conclusions

To our knowledge, this is the first study reporting that 1,5-AG affects glycolysis and leukemia cell growth/survival. After examining a large sample of metabolomics in patients with pre-B ALL, we concluded that 1,5-AG can be considered a pre-B ALL biomarker. Antioxidant drugs provided a possible therapeutic direction for the clinical treatment of pre-B ALL children with higher 1,5-AG.

## Supplementary Information

**Additional file 1: Supplementary data for some experiments. Fig. S1.** Identification of 1,5-AG metabolite by liquid chromatography and mass spectrometry (LC-MS). **Fig. S2.** Effects of different concentrations of vitamin C on pre-B ALL cell viability (**A**) and apoptosis (**B**). 0.2 mM vitamin C was chosen as the intervention concentration. (**C**) Effects of 1,5-AG with 0.2 mM Vitamin C on cell cycle. Data are represented as mean ± SD; *p < 0.05, **p < 0.01, ***p < 0.001. **Fig. S3.** The infiltration of leukemia cells in BM (**A**) and spleen (**B**) for different groups on day 7 was analyzed by flow cytometry. Data were shown as mean ± SD (*n* = 5/group); ns (*p* ≥ 0.05); *p < 0.05; **p < 0.01. **Table S1**. Basic clinical characteristics of the patients.

**Additional file 2.** Full-length original western blots of PDK4 protein and MAPK/ERK-related protein was detected by western blot analysis include multiple exposures (Reh and HAL-01 cell lines).

## Data Availability

The RNA-seq data generated and analysed during the current study are available in the [GEO] repository, [GSE215116] (https://www.ncbi.nlm.nih.gov/geo/query/acc.cgi?acc=GSE215116). Other data will be obtained from the corresponding authors upon reasonable request.

## Abbreviations

1,5-AG: 1,5-anhydroglucitol
Pre-B ALL: pre-B acute lymphoblastic leukemia
AML: acute myeloid leukemia
OPLS-DA: orthogonal partial least squares discriminant analysis
PB: peripheral blood
PB-MNCs: peripheral blood-mononuclear cells
BM: bone marrow
PBS: phosphate-buffered saline
CCK-8: Cell Counting Kit-8
PI: propidium iodide
DCFH-DA: dichloro-dihydro-fluorescein diacetate
NADPH: nicotinamide adenine dinucleotide phosphate
GAPDH: glyceraldehyde 3-phosphate dehydrogenase
IVIS: In Vivo Imaging System
LC-MS: liquid chromatography-mass spectrometry
OCR: Oxygen consumption rates
ECAR: extracellular acidification rate
ROS: reactive oxygen species
MAPK/ERK: mitogen-activated protein kinase/extracellular signal-regulated kinase
NAC: N-acetylcysteine
VC: Vitamin C
