# Supplementary material for: 1,5-Anhydroglucitol promotes pre-B acute lymphocytic leukemia progression by driving glycolysis and reactive oxygen species formation
Source: BMC Cancer. 2023 Feb 6;23:122. doi: 10.1186/s12885-023-10589-9 (PMC9903573; doi:10.1186/s12885-023-10589-9)
Supplement: Supplementary file 1 — Additional file 1: Supplementary data for some experiments. Fig. S1. Identification of 1,5-AG metabolite by liquid chromatography and mass spectrometry (LC-MS). Fig. S2. Effects of different concentrations of vitamin C on pre-B ALL cell viability (A) and apoptosis (B). 0.2 mM vitamin C was chosen as the intervention concentration. (C) Effects of 1,5-AG with 0.2 mM Vitamin C on cell cycle. Data are represented as mean ± SD; *p < 0.05, **p < 0.01, ***p < 0.001. Fig. S3. The infiltration of leukemia cells in BM (A) and spleen (B) for different groups on day 7 was analyzed by flow cytometry. Data were shown as mean ± SD (n = 5/group); ns (p ≥ 0.05); *p < 0.05; **p < 0.01. Table S1. Basic clinical characteristics of the patients. [file 12885_2023_10589_MOESM1_ESM.pdf]

Additional file 1. Supplementary data for some experiments.

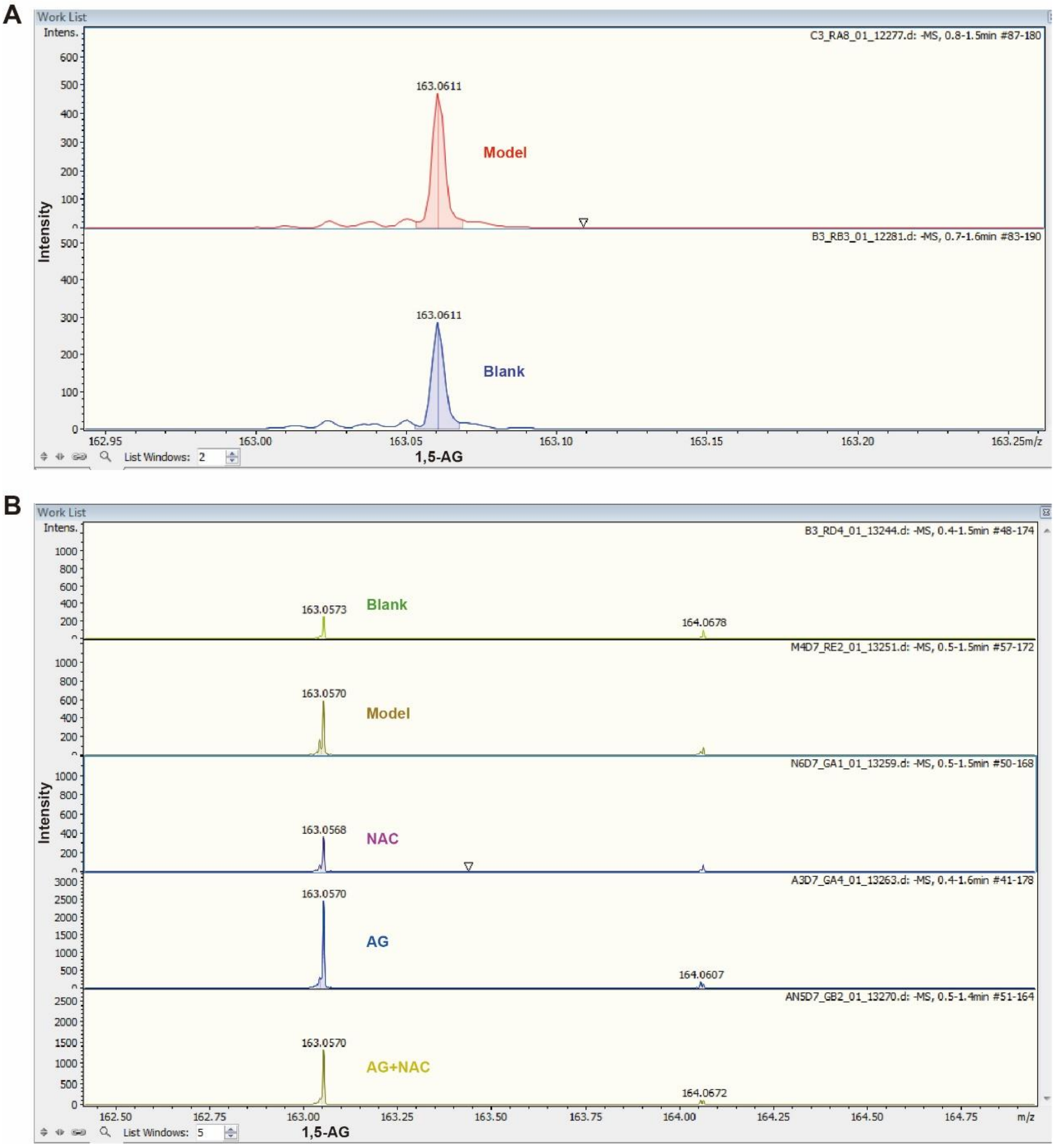

FigS1. Identification of 1,5-AG metabolite by liquid chromatography and mass spectrometry (LC-MS).

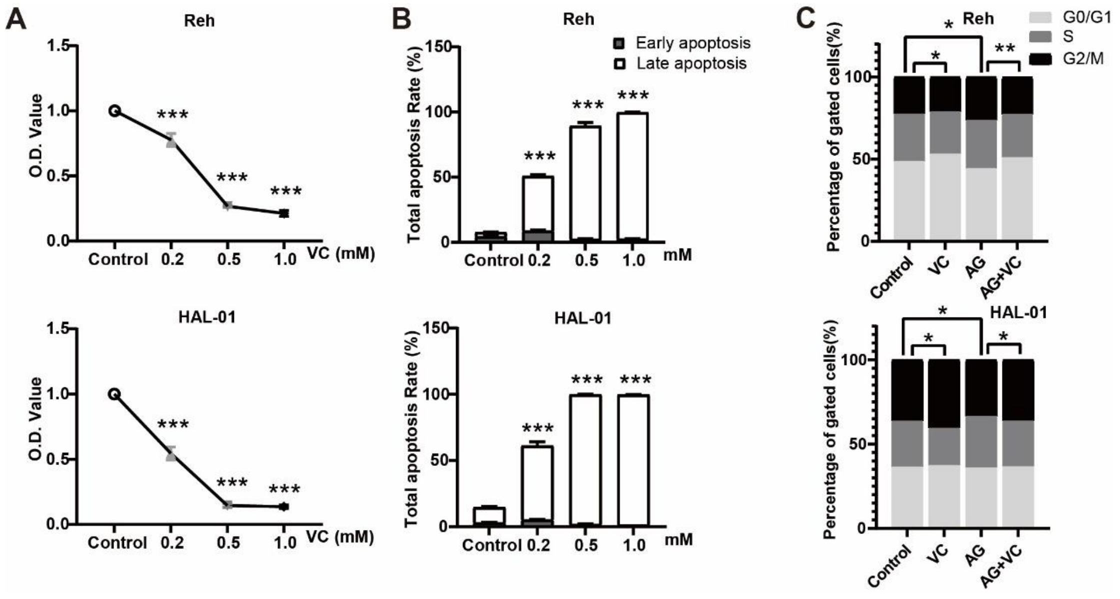

FigS2. Effects of different concentrations of vitamin C on pre-B ALL cell viability (**A**) and apoptosis (**B**). 0.2 mM vitamin C was chosen as the intervention concentration. (**C**) Effects of 1,5-AG with 0.2 mM Vitamin C on cell cycle. Data are represented as mean ± SD; $^{*}p < 0.05$, $^{**}p < 0.01$, $^{***}p < 0.001$.

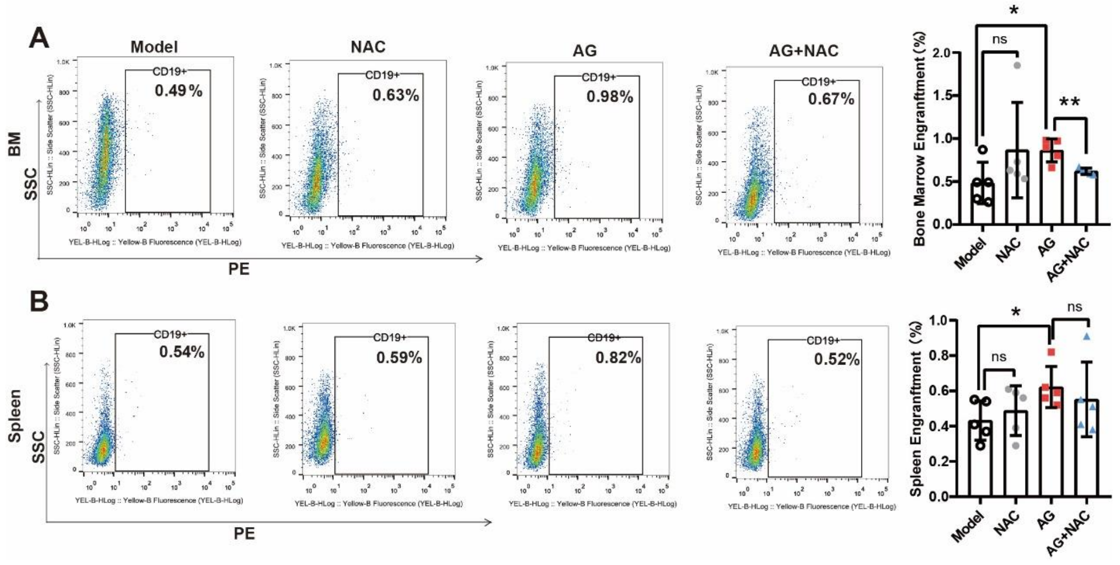

FigS3. The infiltration of leukemia cells in BM (**A**) and spleen(**B**) for different groups on day 7 was analyzed by flow cytometry. Data were shown as mean ± SD (n = 5/group); ns ($p \geq 0.05$); $^{*}p < 0.05$; $^{**}p < 0.01$.

Table S1. Basic clinical characteristics of the patients

	**Pre-B ALL**	**Healthy donor**
Age (months), median	48	42
Gender, male/female	17/10	18/9
Leucocyte count ($\times 10^9$/L), <50/≥50	20/7	--
