# Supplementary material for: 1,5-Anhydroglucitol promotes pre-B acute lymphocytic leukemia progression by driving glycolysis and reactive oxygen species formation
Source: BMC Cancer. 2023 Feb 6;23:122. doi: 10.1186/s12885-023-10589-9 (PMC9903573; doi:10.1186/s12885-023-10589-9)
Supplement: Supplementary file 2 — Additional file 2. Full-length original western blots of PDK4 protein and MAPK/ERK-related protein was detected by western blot analysis include multiple exposures (Reh and HAL-01 cell lines). [file 12885_2023_10589_MOESM2_ESM.pdf]

Additional file 2.

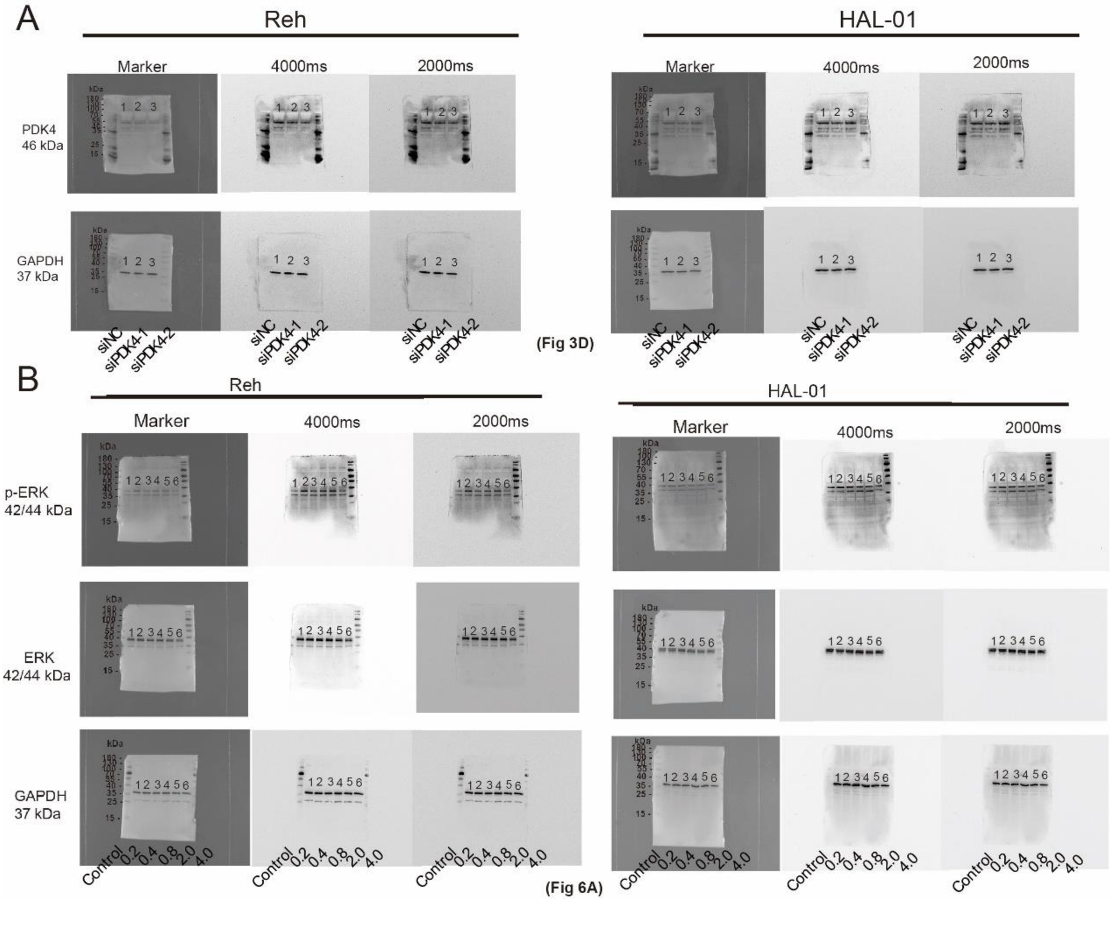

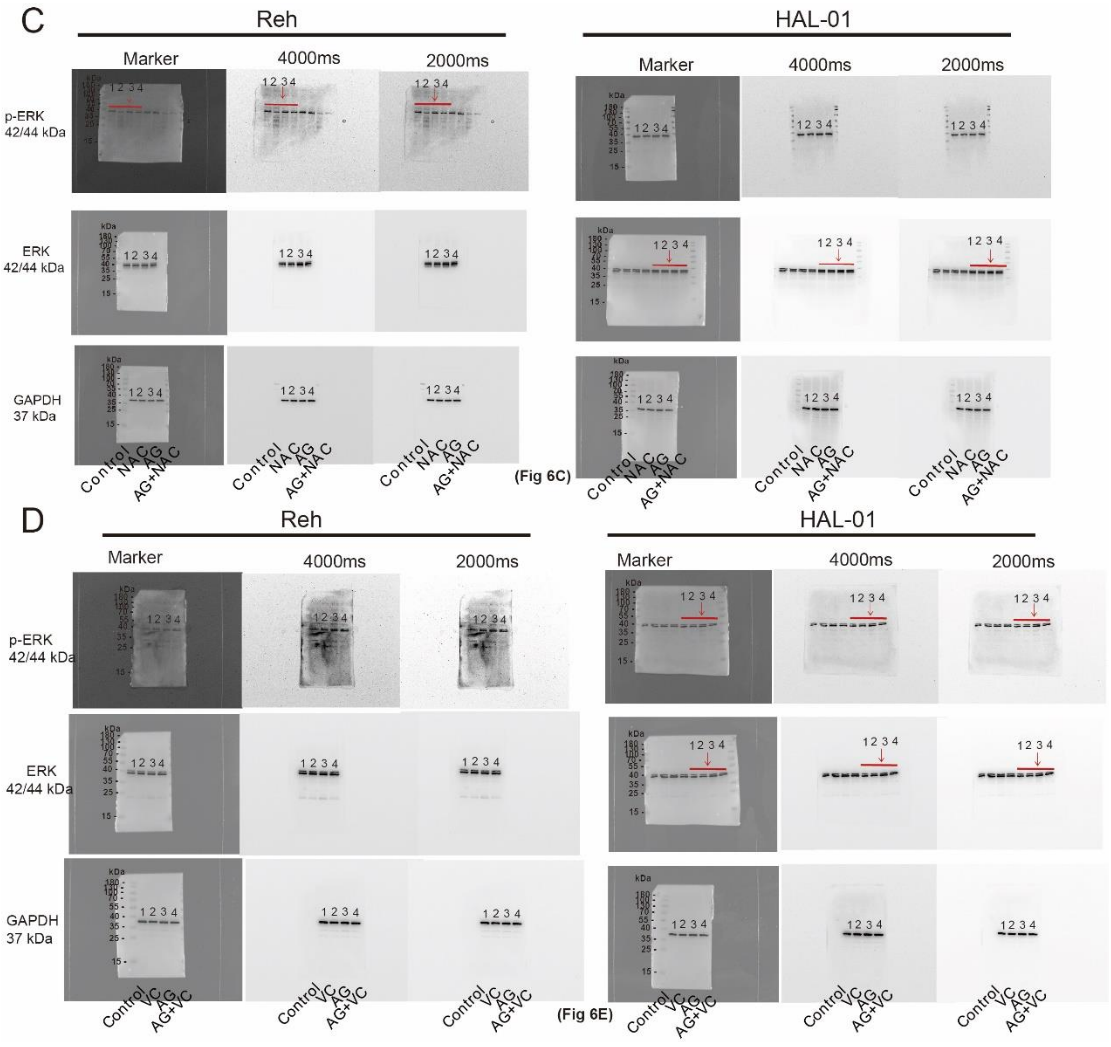

Full-length original western blots of PDK4 protein and MAPK/ERK-related protein was detected by western blot analysis include multiple exposures (Reh and HAL-01 cell lines). (**A**) The original western blot images of knockdown efficiency of PDK4 gene of two different siRNA. (**B**) Original western blot images of the expression levels of p-ERK1/2 and ERK1/2 protein after the intervention of different dose of 1,5-AG. (**C**) . Original western blot images of the expression levels of p-ERK1/2 and ERK1/2 protein after the reverse with NAC. (**D**) Original western blot images of the expression levels of p-ERK1/2 and ERK1/2 protein after the reverse with VC. All the blots were intact and uncut to hybridization with respective antibodies. The samples derive from the same experiment and that blots were processed in parallel.
